# SSLpheno: a self-supervised learning approach for gene–phenotype association prediction using protein–protein interactions and gene ontology data

**DOI:** 10.1093/bioinformatics/btad662

**Published:** 2023-11-06

**Authors:** Xuehua Bi, Weiyang Liang, Qichang Zhao, Jianxin Wang

**Affiliations:** Hunan Provincial Key Lab on Bioinformatics, School of Computer Science and Engineering, Central South University, Changsha 410083, China; Medical Engineering and Technology College, Xinjiang Medical University, Urumqi 830017, China; College of Information Science and Engineering, Xinjiang University, Urumqi 830046, China; Hunan Provincial Key Lab on Bioinformatics, School of Computer Science and Engineering, Central South University, Changsha 410083, China; Hunan Provincial Key Lab on Bioinformatics, School of Computer Science and Engineering, Central South University, Changsha 410083, China

## Abstract

**Motivation:**

Medical genomics faces significant challenges in interpreting disease phenotype and genetic heterogeneity. Despite the establishment of standardized disease phenotype databases, computational methods for predicting gene–phenotype associations still suffer from imbalanced category distribution and a lack of labeled data in small categories.

**Results:**

To address the problem of labeled-data scarcity, we propose a self-supervised learning strategy for gene–phenotype association prediction, called SSLpheno. Our approach utilizes an attributed network that integrates protein–protein interactions and gene ontology data. We apply a Laplacian-based filter to ensure feature smoothness and use self-supervised training to optimize node feature representation. Specifically, we calculate the cosine similarity of feature vectors and select positive and negative sample nodes for reconstruction training labels. We employ a deep neural network for multi-label classification of phenotypes in the downstream task. Our experimental results demonstrate that SSLpheno outperforms state-of-the-art methods, especially in categories with fewer annotations. Moreover, our case studies illustrate the potential of SSLpheno as an effective prescreening tool for gene–phenotype association identification.

**Availability and implementation:**

https://github.com/bixuehua/SSLpheno.

## 1 Introduction

In clinical practice, phenotypes are used to describe deviations from normal morphology, physiology, or behavior (Robinson 2012). Clinicians use phenotypes to identify abnormal conditions through historical or examination data. From a genetics perspective, genes are closely related to the formation of disease phenotype. In recent years, a significant number of candidate genes linked with disease phenotypes have been discovered (Bastarache *et al.* 2022, Horowitz *et al.* 2022). These discoveries have greatly improved our understanding of the genetic basis of diseases, as well as the development of new drugs and novel methods for disease treatment. However, the connection between genes and phenotypes is not straightforward. Typically, mutations in the same gene can cause multiple syndromes, and mutations in different genes can cause the same disorder (Oti and Brunner 2007). Therefore, significant challenges remain in uncovering the links between genes and phenotypes.

Traditional methods for locating associations between genes and phenotypes, such as linkage mapping and genome-wide association studies (GWAS) (Beck *et al.* 2020), are based on the hypothesis of “common disease, common variation,” which does not account for all disease risk. With the proposal of the Human Phenome Project, standardized human phenotypes databases, such as the Human Phenotype Ontology (HPO) (Köhler *et al.* 2021), have facilitated the development of computational methods for predicting gene–phenotype associations (Xue *et al.* 2019a, Chen *et al.* 2021, Yuan *et al.* 2022b). These methods use large amounts of phenotypes and gene-related data and employ various algorithms to predict disease-causing genes, showing promising performance.

Numerous studies (Köhler *et al.* 2009, Hoehndorf *et al.* 2011, Smedley *et al.* 2013) have attempted to establish gene–phenotype associations by calculating the phenotype similarity, which are based on the hypothesis that similar or interacting genes are related with similar phenotypes. However, these approaches are limited due to the incompleteness of known gene–phenotype associations. Gene–phenotype data from model organisms can compensate for lack of human data and increase genome coverage. Researchers have looked to comparing phenotype traits across species to predict the human gene–phenotype associations (Washington *et al.* 2009, Wang *et al.* 2013, Bone *et al.* 2016). The use of model organisms can expand the range of candidate genes, but only genetic and phenotypic information from direct homologs and similar anatomical structures can provide valuable reference (Alghamdi *et al.* 2022). Anyway, these similarity-based methods have produced multiple similarity measures (Kulmanov *et al.* 2021), which provide diverse ideas for the constructing similarity networks in network-based methods.

Information propagation methods, such as label propagation or random walking methods, rely on constructed network to predict gene–phenotype associations (Xie *et al.* 2015, Petegrosso *et al.* 2017). Gene–gene associations (GGAs) are typically represented by genes as nodes and functional associations as edges, which can be described by gene co-expression network (Luo *et al.* 2007) and genetic interaction (Hu *et al.* 2021). Protein–protein interaction (PPI) networks are important types of GGAs that capture gene relationships (Xiang *et al.* 2022). The quality of the constructed network and its construction method can affect the prediction performance of the model, as false positive data can introduce additional bias to both the protein network and phenotype similarity matrix (Ranea *et al.* 2022). Moreover, Dual Label Propagation (DLP) was introduced to predict the associations of genes with their most specific annotated phenotypes in HPO, by simultaneously reconstructing GO term–gene associations and HPO phenotype–gene associations (Petegrosso *et al.* 2017). However, these methods may not be able to predict the phenotypic annotation of isolated points in the network.

Deep learning has also shown powerful capability in bioinformatics due to the ability to automatically extract features from complex biological data (Peng *et al.* 2021, Yuan *et al.* 2022a, Zhang *et al.* 2022). This technology has been widely applied in various areas of bioinformatics, including the analysis of genes and diseases from different omics data, such as genome (Yuan and Bar-Joseph 2019), transcriptome (Wang *et al.* 2023b), proteome (Senior *et al.* 2020), and metabolome (Wang *et al.* 2022a). Researchers have applied deep learning to predict gene–phenotype associations (Kulmanov and Hoehndorf 2020, Pourreza Shahri and Kahanda 2021) and regard gene–phenotype prediction as a multi-label classification task, using GGA network or gene functional information to predict phenotypes. For example, Kulmanov and Hoehndorf (2020) developed DeepPheno, a neural network-based hierarchical multi-label classification method for predicting abnormal phenotypes of a group of genes with missing functional annotations. Pourreza Shahri *et al.* (Pourreza Shahri and Kahanda 2021) proposed a deep semi-supervised fusion framework PPPredSS, which combined deep neural networks (DNNs), semi-supervised and integrated learning to predict protein–phenotype relationships in extended unlabeled datasets based on a small labeled dataset. Since phenotypes arise from molecular network aberrations and physiological interactions within and between cells, tissues, and organs, knowledge about molecular interactions is crucial. GNN-based prediction model, such as HPOFiller (Liu *et al.* 2021), HPODNets (Liu *et al.* 2022b), and GraphPheno (Liu *et al.* 2022c), have a strong advantage in predicting phenotypes from molecular networks. HPOFiller constructed a PPI network, an HPO similarity network and a protein–phenotype bipartite graph, and then iteratively redefined the network representation in each of the three networks with GCN to obtain the protein–phenotype relationships. HPODNets learned the high-order information of proteins in the multi-source PPI networks by using eight GCNs, and then fused the low-dimensional vectors to obtain the HPO prediction results in an end-to-end manner. GraphPheno integrated human protein sequences and PPIs and employed Graph Autoencoder to identify novel protein–phenotype associations.

Although deep learning methods have made significant progress in predicting gene–phenotype associations, these methods require large-scale labeled data for training. The main challenge remains that the role of a large number genes for abnormal phenotypes is still unknown. The HPO database as of March 2023 has only annotated phenotype for 4895 genes. This number is relatively small compared to the 25 000 human genes, which hinders the performance improvement of supervised deep learning in gene–phenotype association prediction.

To address the scarcity of labeled data, self-supervised learning (SSL) methods have been introduced for many bioinformatics tasks, such as molecular property prediction (Zheng *et al.* 2023), compound–protein affinity and contact prediction (You and Shen 2022). Recently, SSL on graphs has also made great progress (Liu *et al.* 2022d). Graph contrastive learning (GCL) utilizes the data augmentation methods to learn feature embedding of graphs (Qiu *et al.* 2020). KAUR (Ma *et al.* 2023) proposed a knowledge-based GCL, regarding nodes and their semantic neighbor information as positive contrastive pairs to enhance the node representations. CasANGCL (Zheng *et al.* 2023) adopted three augmentation methods, attribute masking, edge perturbation, and subgraph extraction, to construct positive and negative samples for molecular property prediction. ATMOL (Liu *et al.* 2022a) constructed an attention-wise masking module by masking the nodes with high attention weights to produce the augmented view. The performance of GCLs heavily relies on well-designed data augmentation strategies. Therefore, it is crucial to design an appropriate augmentation method for SSL.

**Figure 1.**
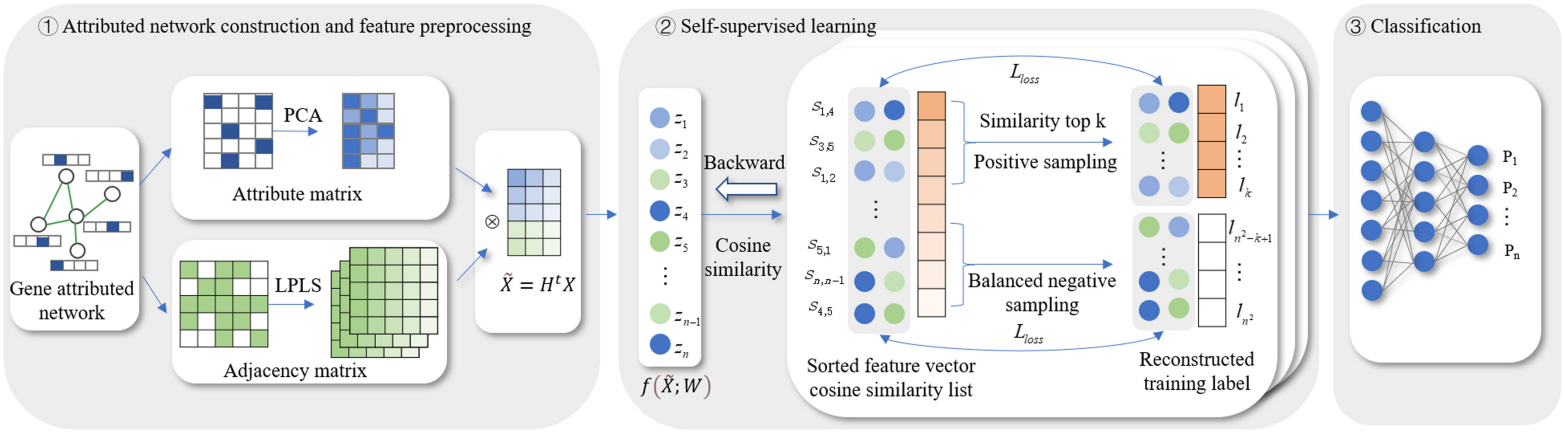
The workflow of SSLpheno. ① Attributed network construction and feature preprocessing. ② Self-supervised learning for pre-training. ③ Multi-label classification for phenotype prediction.

Inspired by these SSL methods, we introduce an SSL-based pre-training model, named SSLpheno, to improve the gene–phenotype association prediction. The detailed process of SSLpheno is shown in Fig. 1. Firstly, by integrating PPIs and gene ontology, we construct a gene attributed network for pre-training, and design a filter to smooth the nodes features. Next, we generate the labels of the SSL training set by computing the cosine similarity of the preprocessed node features. During training, we iteratively select the set of positive and negative samples to enhance the feature representation of nodes. Finally, we use supervised learning DNN to predict the gene–phenotype associations by multi-label classification. Experimental results demonstrate that our SSLpheno model outperforms state-of-the-art methods for gene–phenotype association prediction. Ablation study and experiments under GGAs show that the design of SSLpheno is reasonable. We also conduct case studies on the phenotype and gene sets predicted by our model. The results indicate the practical value of SSLpheno in gene–phenotype association prediction.

## 2 Materials and methods

### 2.1 Datasets

We utilize two datasets to facilitate model comparison, which are constructed from the HPO (Schriml *et al.* 2022) and the DisGeNET (Piñero *et al.* 2020). The HPO database provides ontologies of human disease phenotype and reliable associations between genes and phenotypes. We download the gene–phenotype associations released on 21 August 2020, which contains 4455 genes, 8093 phenotypes, and 176 559 annotations from https://hpo.jax.org. Since the directed acyclic graph structure of the phenotype ontology follows the true-path-rule, we perform annotation propagation on the obtained gene–phenotype relationships. To map genes to proteins, we use the mapping tool in Uniport/Swissport (https://www.uniprot.org/id-mapping) and select the genes that map to manually reviewed and annotated proteins. Then, we retain the terms in the Phenotype Abnormal branch of the phenotype ontology followed the previous works (Kulmanov and Hoehndorf 2020, Liu *et al.* 2021) to ensure the fairness of the comparison with the baseline models. Furthermore, we remove terms with annotation counts of ≤10, similar to Graphpheno, HPODNets, and HPOFiller, which is the partial evaluation mode proposed by CAFA2 (Jiang *et al.* 2016) to ensure the model stability. In total, we get a dataset, referred to as “HPO_2020,” containing 3709 genes, 3895 phenotypes and 525 056 annotations with an average of 141.6 annotations per gene. We divide the phenotype terms in the dataset into four groups, according to the number of annotated genes: “11–30,” “31–100,” “101–300,” and “>300” (Xue *et al.* 2019b, Liu *et al.* 2022b). DisGeNET is a platform providing abundant information about human disease-associated genes and variants. By using the disgenet2r package (Piñero *et al.* 2020), we filter data where the disease type is “phenotype” to generate gene–phenotype association dataset. At last, we get 1 657 434 gene–phenotype associations after the same preprocessing as for the “HPO_2020,” involving 15 271 genes and 4755 phenotypes with an average of 108.5 annotations per gene. Table 1 shows the statistic of the two datasets, grouped by the number of genes annotated with each phenotype.

**Table 1.** Statistic of genes, phenotype terms, and annotations in datasets.

Datasets	Genes	Phenotype terms				Avg. annotations per gene
		11–30	31–100	101–300	≥301	
HPO_2020	3709	1624	1235	729	441	141.6
DisGeNET	15 271	1552	1370	879	954	108.5

### 2.2 Proposed methods

SSLpheno is constructed followed by three steps: Firstly, we construct the gene attributed network with PPIs and gene ontology, and design a filter based on Laplacian smoothing filter to denoise the high-frequency components of the feature matrix. In the second step, SSL is used for pre-training, during which rich feature representations are learned through a well-designed pretext task to provide more generalized gene feature for the downstream task. We finally feed the pre-trained features into a multi-layer neural network to predict phenotypes.

#### 2.2.1 Attributed network construction and feature preprocessing

Genes and their products participate in various life activities of organisms mainly through direct interaction or indirect synergistic interaction, collectively known as GGAs (Guala *et al.* 2020). In this study, we construct GGAs by using PPIs from STRING (Szklarczyk *et al.* 2021) database (https://string-db.org/) (v11.0). The “combined score” provided by STRING is scaled to [0,1]. Following the works (Fan *et al.* 2020, Liu *et al.* 2022c), we set the edge between their mapped genes to 1 if the score of two proteins is not <0.3, and 0 otherwise, to ensure that we only use highly confident interactions. Considering that functionally related genes produce similar phenotypes (Claussnitzer *et al.* 2020), we use GO annotations as the attributes of nodes in the GGAs. The GO annotations are obtained from Uniport/Swissport and comprised 13 778 classes. We encoded the GO terms with a 13 778 dimensional binarized vector, where the *i*-th bit being 1 indicates having the *i*-th GO annotation. Considering the difficulty of high-dimensional sparse feature in graph representation learning, we use principal component analysis (PCA) to reduce the dimensionality and eliminate data sparsity. Finally, we obtain the attributed network used in this study, which is formally defined as: G={V,E,X}, where $V=\left\{v_{1},v_{2},\ldots ,v_{n}\right\}$ is nodes set of the graph, $v_{i}$ denotes gene *i* and *n* is the number of nodes in the graph. *E* is the edges set, and each edge represents the relationship between two genes. $X={\left[x_{1},x_{2},\ldots ,x_{n}\right]}^{T}$ is the attribute matrix, where $x_{i}\in R^{m}$ denotes the attribute representation of gene node $v_{i}$. The adjacency matrix can be defined as $A=\left\{a_{ij}|1\leq i,j\leq n\right\}\in R^{2}$, where $a_{ij}=1$ if $(v_{i},v_{j})\in E$. After PCA processing, the attribute original dimension *m* is reduced from 13 778 to 1000, which is optimal by experimental verification, as shown in Supplementary Fig. S5.

Given the constructed attributed network, a feature filter based on Laplacian is designed for feature smoothing. The embedding of nodes in the attributed graph should consider both the similarity of node attributes and the consistency of graph topology (Huang *et al.* 2017). To preserve self-information, we define the adjacency matrix with added self-connections as $\tilde{A}=I+A$, where *I* is the identity matrix. $\tilde{D}=\mathrm{diag}({\tilde{d}}_{1},{\tilde{d}}_{2},\ldots ,{\tilde{d}}_{n})\in R^{n\times n}$ denotes the degree matrix of $\tilde{A}$, where ${\tilde{d}}_{i}=\sum_{v_{j}\in V}{\tilde{a}}_{ij}$ is the degree of node $v_{i}$. $\tilde{L}=\tilde{D}+\tilde{A}$ is Laplacian matrix corresponding to $\tilde{A}$. We employ the symmetric normalized graph Laplacian, ${\tilde{L}}_{\mathrm{sym}}={\tilde{D}}^{-\frac{1}{2}}\tilde{L}{\tilde{D}}^{-\frac{1}{2}}$. The filter based on generalized Laplacian smoothing filter is defined as


(1) $$H=I-\alpha {\tilde{L}}_{\mathrm{sym}}.$$

There are different choices for α in different works (Wang *et al.* 2019, Cui *et al.* 2020), we set α=2/3 according to the experimental results shown in Supplementary Fig. S6. After preprocessing the gene feature matrix through *t* defined filters, we obtained the filtered feature matrix as


(2) $$\tilde{X}=H^{t}X.$$

#### 2.2.2 SSL for pre-training

The purpose of pre-training is to generate enhanced low-dimensional embedding representations for nodes in graph (Hu *et al.* 2019). Given the filtered node features $\tilde{X}$, we encode the node embeddings using a linear encoder


(3) $$f:Z=f(\tilde{X};W)=\tilde{X}W,$$

where *W* is the weight matrix. The min. to max. scaler is used to scale the embeddings to [0,1].

The adjacency matrix is usually chosen as the real label of node pairs in Graph Auto Encoder (GAE) (Kipf and Welling 2016). However, this method only records one-hop structure information, which is insufficient for attributed graph embedding. Inspired by the Adaptive Graph Encoder (Cui *et al.* 2020), we define the labels of the gene pairs using their similarity. Specifically, we first compute the cosine similarity of gene pairs $S=\left\{s_{ij}|1\leq i,j\leq n\right\}\in R^{n^{2}}$, which is defined as


(4) $$S=\frac{ZZ^{T}}{{\left\|Z\right\|}_{2}^{2}},$$

where *Z* is the gene features after encoding by the linear encoder, which comes from Equation (3). Then, we rank the similarity sequence and select positive and negative samples from it for training. We select the top *k* gene pairs $\left(v_{i},v_{j}\right)$ with the highest similarity as positive samples and *k* pairs of genes with the lowest similarity value are selected as negative samples. Finally, the training set consisting of 2*k* gene pairs are obtained.

To train the gene pairs auto-encoder, we used cross-entropy as the loss function:


(5) $$L_{\mathrm{self}}=-\sum_{(v_{i},v_{j})\in M}[y_{\mathrm{self}}\;\text{log}(s_{ij})+(1-y_{\mathrm{self}})\log(1-s_{ij})],$$

where $y_{\mathrm{self}}$ is the training label of the gene pair $\left(v_{i},v_{j}\right)$, which is set to 1 if the gene pair is positive, and 0 if the gene pair is negative, and $s_{ij}$ is the similarity of the gene pair $\left(v_{i},v_{j}\right)$. After the SSL, we get the final gene representations $Z=\left\{z_{1},z_{2},\ldots ,z_{n}\right\}\in R^{n}$, where $z_{i}$ is the trained feature vector of gene $v_{i}$, and its dimension is experimentally set to 2048. The details are shown in Supplementary Fig. S7.

#### 2.2.3 DNN classifier for prediction

DNN has powerful performance on supervised learning tasks (LeCun *et al.* 2015). We use DNN as the classifier for predicting phenotype. The input is the gene feature vectors trained by the pre-trained model, with three hidden layers are stacked between the input and output layers. The output layer consists of 3895 phenotype classes. In each layer, we first perform a dense function f(⋅) and Batch Normalization on the input features, and then conduct LeakyReLU activation. Each layer is defined as


(6) $$Z^{l+1}=\mathrm{LeakyReLU}(BN(f(Z^{l}))),$$

where $Z^{l}$ is the input of the *l*-th layer, and $Z^{\left(l+1\right)}$ is the output of the *l*-th layer.

For the last output layer, we use a sigmoid activation function σ(⋅). During training, the gradient descent algorithm is used, along with the cross-entropy function. The DNN classifier loss function to be optimized is as follows


(7) $$L_{\mathrm{cla}}=-\sum [y_{\mathrm{cla}}\;\text{log}(p)+(1-y_{\mathrm{cla}})\log(1-p)],$$

where $y_{\mathrm{cla}}$ is the label of gene in training set and *p* is the predicted probability value. We use a batch size of 128 and an initial learning rate 0.001.

### 2.3 Parameters

SSLpheno has been implemented using PyTorch and trained using the Adam optimizer. We utilize an empirical and heuristic approaches for hyper-parameter tuning through grid-search. The default hyper-parameter settings, the search scopes and the optimized values are summarized in Supplementary Table S2. For all subsequent experiments, we adapt the optimal hyper-parameter settings.

## 3 Results and discussion

### 3.1 Evaluation criteria

In this work, we employ the area under the precision–recall curve (AUPR), *F*1 score (*F*1) as evaluation metrics. They are the commonly used metrics for the gene–phenotype association prediction (Kulmanov and Hoehndorf 2020, Liu *et al.* 2021, 2022b).

Due to the imbalanced class problems in the experimental datasets, we evaluate the prediction performance under two scenarios, similar to previous works (Liu *et al.* 2022b): (i) macro-averaged, denoted as M, where metrics are calculated for each term, and the average is determined, and (ii) micro-averaged, denoted as m, where the predicted score matrix for each gene–phenotype term pair is vectorized, and metrics are calculated based on the resulting vector. The best result is displayed in bold, while the second-best is underlined.

### 3.2 Prediction performance with baseline methods

We have compared our method with six state-of-the-art baseline methods, including deep learning-based methods GraphPheno (Kulmanov and Hoehndorf 2020), HPODNets (Liu *et al.* 2022b), and HPOFiller (Liu *et al.* 2021), two classical methods tlDLP (Petegrosso *et al.* 2017) and BiRW (Xie *et al.* 2015) for gene–phenotype association prediction, and another method proposed by ourselves employing GAE for pre-training. To demonstrate the models’ generalization ability, we compare the prediction performance on the two datasets mentioned in Section 2.1. In order to fairly compare SSLpheno and the baseline methods, we use a 5-fold cross-validation (CV) for evaluation. We obtain and preprocess the data of baseline methods according to their paper’s description.

Prediction performance comparison results between SSLpheno and baseline methods for macro-averaged metrics are presented in Table 2, while those for micro-averaged metrics are shown in Table 3. Generally, SSLpheno achieves superior results compared to baseline methods on both datasets HPO and DisGeNET. SSLpheno_GAE and SSLpheno adopting strategy pre-training achieve better results than HPODNets and HPOFiller, which adopt end-to-end training. This indicates that the pre-training strategy can alleviate the impact of data sparsity on model performance in gene–phenotype prediction problem. Additionally, compared with GraphPheno, which adopted sequence features, our model adopts GO as features and achieves better results. Furthermore, the methods based on graph, HPODNets and HPOFiller, basically outperform the network propagation-based methods tlDLP and BiRW. It is worth mentioning that BiRW utilized GO as a cross-domain knowledge source take a better performance than GraphPheno. It also illustrates that GO contributes to the prediction of gene–phenotype associations. These experimental results indicate that the SSL strategy our model employed is more suitable for predicting gene–phenotype associations. On the dataset DisGeNET, which is shown in Supplementary Fig. S1 to have extremely imbalanced phenotype annotation distribution, our model can still achieve the best prediction performance.

**Table 2.** Performance of CV under macro-averaged metrics.

Datasets	Methods	Term (11–30)		Term (31–100)		Term (101–300)		Term (>300)		All	
		AUPR	$F_{\max}$	AUPR	$F_{\max}$	AUPR	$F_{\max}$	AUPR	$F_{\max}$	AUPR	$F_{\max}$
HPO_2020	HPODNets	0.290	0.383	0.318	0.410	0.360	0.418	**0.538**	**0.531**	0.337	0.413
	GraphPheno	0.167	0.158	0.191	0.217	0.230	0.260	0.423	0.518	0.206	0.326
	HPOFiller	0.257	0.265	0.271	0.300	0.312	0.332	0.494	0.503	0.288	0.356
	tlDLP	0.141	0.147	0.156	0.192	0.197	0.242	0.388	0.494	0.174	0.383
	BiRW	0.173	0.187	0.193	0.225	0.245	0.281	0.440	0.520	0.211	0.411
	SSLpheno_GAE	0.299	0.385	0.322	0.414	0.350	0.416	0.504	0.521	0.337	0.414
	SSLpheno	**0.332**	**0.402**	**0.339**	**0.415**	**0.370**	**0.432**	0.529	**0.531**	**0.350**	**0.427**
DisGeNET	HPODNets	0.200	0.288	0.216	0.318	0.247	0.333	**0.404**	**0.431**	0.254	0.334
	GraphPheno	0.156	0.231	0.177	0.279	0.132	0.256	0.313	0.339	0.191	0.299
	HPOFiller	0.063	0.125	0.090	0.174	0.149	0.236	0.331	0.377	0.113	0.185
	tlDLP	0.165	0.221	0.187	0.276	0.200	0.299	0.365	0.400	0.201	0.288
	BiRW	0.088	0.200	0.208	0.281	0.187	0.293	0.356	0.377	0.199	0.301
	SSLpheno_GAE	0.211	0.291	0.184	0.296	0.226	0.302	0.377	0.414	0.225	0.303
	SSLpheno	**0.225**	**0.292**	**0.243**	**0.342**	**0.255**	**0.343**	0.407	**0.436**	**0.275**	**0.348**

**Table 3.** Performance of CV under micro-averaged metrics.

Datasets	Methods	Term (11–30)		Term (31–100)		Term (101–300)		Term (>300)		All	
		AUPR	$F_{\max}$	AUPR	$F_{\max}$	AUPR	$F_{\max}$	AUPR	$F_{\max}$	AUPR	$F_{\max}$
HPO_2020	HPODNets	0.203	0.283	0.289	0.342	**0.351**	0.379	0.585	0.548	0.412	0.441
	Graphpheno	0.135	0.213	0.153	0.224	0.219	0.268	0.533	0.545	0.396	0.422
	HPOFiller	0.189	0.255	0.244	0.301	0.306	0.342	0.573	0.556	0.435	0.453
	tlDLP	0.071	0.136	0.119	0.245	0.179	0.287	0.470	0.510	0.310	0.389
	BiRW	0.112	0.201	0.165	0.263	0.234	0.284	0.521	0.536	0.363	0.407
	SSLpheno_GAE	0.225	0.305	0.291	**0.352**	0.335	0.379	0.589	0.574	0.474	0.496
	SSLpheno	**0.250**	**0.330**	**0.297**	0.350	0.336	**0.386**	**0.596**	**0.576**	**0.484**	**0.505**
DisGeNET	HPODNets	0.121	0.206	0.175	**0.257**	0.229	0.295	0.481	0.467	0.339	0.396
	Graphpheno	0.069	0.157	0.112	0.199	0.145	0.236	0.398	0.389	0.478	0.481
	HPOFiller	0.017	0.056	0.050	0.113	0.122	0.198	0.432	0.442	0.313	0.358
	tlDLP	0.073	0.167	0.115	0.204	0.157	0.245	0.403	0.396	0.468	0.470
	BiRW	0.089	0.176	0.144	0.210	0.189	0.255	0.436	0.487	0.453	0.449
	SSLpheno_GAE	0.091	0.190	0.165	0.242	0.177	0.254	0.559	0.527	0.498	0.491
	SSLpheno	**0.136**	**0.213**	**0.180**	0.251	**0.233**	**0.291**	**0.574**	**0.539**	**0.515**	**0.504**

The HPO database provides data versions at different points in time, which we use for temporal validation to assess the performance of our model. The benchmark dataset is created using the method proposed in CAFA2 (Jiang *et al.* 2016), which includes genes that have no annotations in the HPO ontology at time $t_{0}$ but accumulate at least one phenotype term with experimental validation between $t_{0}$ and $t_{1}$. According to this criterion, the training set consists of gene–phenotype associations released by the HPO database on 15 November 2019, while the test set consists of new associations added from 15 November 2019 to 25 August 2020. The distributions of the dataset are shown in Supplementary Fig. S2.

In temporal validation, we also calculate the performance comparison results separately for macro and micro measures, as shown in Tables 4 and 5, respectively. SSLpheno achieves the best results in almost all cases, implying that our model is more powerful for gene–phenotype association prediction with low-annotated data. Temporal validation actually is a form of *de novo* test with entirely worse performance than CV. It may be caused by the incompleteness of annotations. We furtherly discuss the case study of new genes/phenotypes prediction in Section 3.4.

**Table 4.** Performance of temporal validation under macro-averaged metrics.

Methods	Term (11–30)		Term (31–100)		Term (101–300)		Term (>300)		All	
	AUPR	$F_{\max}$	AUPR	$F_{\max}$	AUPR	$F_{\max}$	AUPR	$F_{\max}$	AUPR	$F_{\max}$
HPODNets	0.097	**0.138**	0.084	0.137	0.101	0.170	0.220	0.306	0.105	0.176
Graphpheno	0.075	0.101	0.076	0.125	0.082	0.164	0.199	0.290	0.102	0.165
HPOFiller	0.088	0.103	0.083	0.130	0.097	0.164	0.205	0.299	0.101	0.169
tlDLP	0.065	0.079	0.066	0.106	0.067	0.123	0.155	0.199	0.100	0.144
BiRW	0.073	0.099	0.065	0.104	0.079	0.135	0.164	0.188	0.095	0.144
SSLpheno_GAE	0.101	0.134	0.092	0.147	0.104	0.185	0.211	0.315	0.115	0.175
SSLpheno	**0.108**	**0.138**	**0.093**	**0.150**	**0.111**	**0.189**	**0.244**	**0.324**	**0.121**	**0.182**

**Table 5.** Performance of temporal validation under micro-averaged metrics.

Methods	Term (11–30)		Term (31–100)		Term (101–300)		Term (>300)		All	
	AUPR	$F_{\max}$	AUPR	$F_{\max}$	AUPR	$F_{\max}$	AUPR	$F_{\max}$	AUPR	$F_{\max}$
HPODNets	**0.038**	0.076	0.042	0.090	0.073	0.124	0.281	0.350	0.152	0.240
Graphpheno	0.021	0.063	0.038	0.089	0.062	0.109	0.267	0.336	0.147	0.216
HPOFiller	0.030	0.072	0.040	0.089	0.069	0.116	0.275	0.347	0.150	0.229
tlDLP	0.022	0.063	0.035	0.077	0.054	0.106	0.205	0.268	0.122	0.172
BiRW	0.030	0.068	0.033	0.080	0.055	0.098	0.201	0.256	0.129	0.193
SSLpheno_GAE	0.031	0.072	0.046	0.092	0.072	0.128	0.303	0.367	0.199	0.291
SSLpheno	0.032	**0.079**	**0.047**	**0.093**	**0.075**	**0.129**	**0.312**	**0.373**	**0.213**	**0.300**

### 3.3 Ablation study

#### 3.3.1 Comparison of different components

To evaluate the contribution of each component, we implement models without each individual component for gene–phenotype association prediction. Finally, we compare SSLpheno with different methods, and the experimental results are presented in Table 6. The results show that each component of SSLpheno exhibits superiority in the phenotype prediction task. Specifically, applying a self-supervised pre-training strategy after any of the data preprocessing and feature smoothing methods can lead to a substantial improvement in prediction performance. Moreover, we also observe that the performance of the filter is limited on high-dimensional sparse features, and PCA is an effective feature dimension reduction method for SSLpheno.

**Table 6.** Phenotype prediction performance with different components.

Component	M-aupr	M-$F_{\max}$	m-aupr	m-$F_{\max}$
PCA	0.223	0.295	0.320	0.400
PCA+SSL	0.264	0.333	0.365	0.414
Filter+SSL	0.303	0.382	0.454	0.468
PCA+Filter+SSL	0.350	0.427	0.484	0.505

#### 3.3.2 Comparison of different GGAs

Different GGAs affect the prediction performance of the model (Wang *et al.* 2017). In addition to STRING, we employ two other well-used GGAs networks, HumanNet (Kim *et al.* 2022) and geneMANIA (Franz *et al.* 2018), to evaluate the impact of different GGAs on our model. The construction of the attributed network followed the approach described in Section 2.2.1. We use SSLpheno to make prediction based on GGAs built with the three different PPI networks and compare the results. Furthermore, we concatenate features obtained by pre-training on the three GGAs and feed them into the downstream classifier. The results of prediction performance with different GGAs are shown in Table 7.

**Table 7.** Phenotype prediction performance with different GGAs.

GGAs	M-aupr	M-$F_{\max}$	m-aupr	m-$F_{\max}$
HumanNet	0.314	0.412	0.411	0.462
GeneMANIA	0.207	0.281	0.348	0.409
STRING	0.350	0.427	0.484	0.505
Combined	0.320	0.395	0.460	0.488

The results indicate that SSLpheno with STRING achieves the best performance, while HumanNet has relatively poor performance with M-aupr of 0.306, and GeneMANIA has the lowest performance. This finding highlights the significance of the quality of GGA networks in gene–phenotype association prediction. Although some studies (Liu *et al.* 2020, 2022b) have shown that combining multiple networks can improves the predictive power of their models, our feature merging strategy does not achieve optimal result. In fact, the differences in the results of these three networks are likely due to differences in the methods used for database generation. The associations in STRING database are mainly derived from high-throughput experimental data (Szklarczyk *et al.* 2021), whereas the other two databases are generated by algorithm (Franz *et al.* 2018, Kim *et al.* 2022). Additionally, our findings suggest that simple concatenation of features may introduce noise and negatively impact downstream tasks.

To explore the embeddings produced by SSLpheno with different GGAs, we embed these vector representations into a 2D space using t-SNE and visualize in Supplementary Fig. S4. We highlight the associated genes with HP: 0002206 with red dots. The features from STRING seem to be a little more concentrated than the features from HumanNet. And the features are distributed entirely produced from GeneMANIA. This is also consistent with the results in Table 7. This suggests that the STRING-generated features are sufficiently capable of distinguishing the genes involved.

#### 3.3.3 Comparison of different classifiers

In order to select a high-performance classifier for downstream task, we conduct experiments to compare the performance of different classifiers and evaluate their impact on SSLpheno. Specifically, to ensure a suitable evaluation, we keep the network construction and SSL components of the model unchanged. Apart from the DNN used in our model SSLpheno, we also introduce three commonly used machine-learning methods, which are Support Vector Machine (SVM), Random Forest (RF), and Logistic Regression (LR). Table 8 lists the results of the 5-fold CV performance. As can be seen, DNN achieves the best results under four metrics, and SVM achieves the second rank. It is remarkable that our method outperforms the baselines in Table 2 on all classifiers, which indicates that the pre-training strategy of our model is effective for gene–phenotype association prediction.

**Table 8.** Phenotype prediction performance with different classifiers.

Classifiers	M-aupr	M-$F_{\max}$	m-aupr	m-$F_{\max}$
SVM	0.341	0.379	0.479	0.482
RF	0.326	0.354	0.420	0.447
LR	0.290	0.305	0.389	0.468
DNN	0.350	0.427	0.484	0.505

### 3.4 Case studies

To demonstrate the capability of SSLpheno for predicting gene–phenotype associations in practical applications, we validate its performance by conducting case studies from two perspectives.

#### 3.4.1 Case study on predicted phenotypes related to genes

By comparing the similarity between predicted phenotypes related to genes and disease phenotypes, we can prioritize genetic variations that lead to diseases or further predict the relationships between genes and diseases, thus improving the identification of pathogenic variations in a clinical setting. In this case study, our primary objective is to assess the suitability of the model-generated phenotype annotations for gene–disease association predictions. We select three genes, *CACNA2D1*, *FBXW4*, and *RAB11A* to conduct this case study. *CACNA2D1* is related to 18 phenotypes in the 2020 database version, which has to be proved one of the genes encoding the α2δ “subunit” and its high expression levels is closely associated with a poor prognosis in ovarian cancer, lung cancer, and hepatocellular carcinoma (Inoue *et al.* 2022). *FBXW4*, a newly added gene in the 2023 HPO, is associated with acute myeloid leukemia and serves as a reliable predictor of chemotherapy sensitivity (Zhang *et al.* 2020). *RAB11A* encodes the protein belonging to the Rab family of small GTPase and plays an important role in human carcinogenesis (Wang *et al.* 2022b), which is a *de novo* without phenotype annotation registered in HPO. Each gene of these three has the highest predicted score in its category.

We use Phenomizer (Köhler *et al.* 2009), a popular tool for disease-ranking, to evaluate our predictions. Table 9 lists the candidate diseases recommended by Phenomizer and supported evidences. The prediction results by SSLpheno show that *CACNA2D1* is associated with 36 phenotypes out of 9226 phenotypes. The predicted phenotypes are used to further predict its related diseases, and the disease with the highest prediction score is epilepsy as shown in Table 9. This result is confirmed by Dahimene *et al.* (2022), they demonstrated that biallelic *CACNA2D1* variants cause a phenotypic spectrum ranging from congenital ataxia with cerebellar vermian atrophy on brain imaging to cerebellar atrophy and developmental and epileptic encephalopathies. SSLpheno predicts that *FBXW4* is associated with 235 phenotypes, which is similarity with disease “split hand/foot malformation” (SHFM). Qiu *et al.* (2022) provided evidence by using trio clinical exome sequencing that abnormal *FBXW4* expression can cause SHFM. SSLpheno prediction results to infer that *RAB11A* is associated with mental developmental disorders, however, we have no direct evidence for this in the literature. Only the literature has shown that*RAB11A* is a key gene associated with Parkinson’s disease (Yin *et al.* 2021). This shows it is difficult to make accurate predictions for genes without prior knowledge.

**Table 9.** Gene–disease annotations calculated with the phenotypes predicted by SSLpheno.

Gene	Disease ID	Disease name	Score (*P* < 0.05)	Evidence
CACNA2D1	OMIM: 300491	Epilepsy	2.29	(Dahimene *et al.* 2022)
FBXW4	ORPHANET: 2440	Split hand/split foot malformation	2.47	(Qiu *et al.* 2022)
RAB11A	OMIM: 30080	Mental retardation	0.49	(Hill *et al.* 2019)

#### 3.4.2 False positive analysis of predicted genes related to phenotype terms

Since the known gene–phenotype associations are serious incomplete, some of the false positive we predicted are actually correct associations. We analysis the false positive results predicted by SSLpheno. “Pneumonia” (HP: 0002090) is a clinical symptom of lower respiratory tract infection. Coronavirus disease 2019 is a special disease, which has attracted close attention of scientists in the past 2 years, and “pneumonia” is one of its main clinical phenotypes (Wang *et al.* 2023a). “Pneumonia” is annotated with 88 genes in the HPO database in August 2020. We predict 136 genes with SSLpheno, where 65 genes are true positive while 71 are false positive. Table 10 lists the details of top 15 predicted false positive genes of “Pneumonia” (HP: 0002090). We investigate if the prediction results were reported in the latest HPO version, GWAS studies and PubMed. Eleven of the predicted genes have supported evidence. Three genes among them are newly added in 2023, 4 associations with cases can be found in GWAS Catalog, and 10 of them are related with PubMed literatures.

**Table 10.** The top 15 predicted false positive genes of pneumonia with supporting literature.

Rank	Gene name	Evidence
1	IL12RB1	PMID: 34306143 (Xu *et al.* 2021)
2	HMBS	
3	ACE2	PMID: 35241825 (Horowitz *et al.* 2022)
4	IL-7	PMID: 32728202 (Monneret *et al.* 2020)
5	IREB2	PMID: 33304392 (Zeng *et al.* 2020)
6	HPGD	PMID: 35180297 (Feitosa *et al.* 2022)
7	DNAL4	
8	ANO3	PMID: 35513865 (Tang *et al.* 2022)
9	DAB1	PMID: 33888907 (Shelton *et al.* 2021)
10	KREMEN1	
11	SEC24D	
12	MTM1	HPO 2023
13	FOCAD	PMID: 25637605 (Pouwels *et al.* 2015)
14	TYRP1	PMID: 35910207 (Zecevic *et al.* 2022)
15	IREB2	PMID: 34340725 (Campos *et al.* 2021)

Furthermore, we also conduct analysis on “Abnormality of cardiovascular system morphology” (ACSM, HP: 0030680). ACSM is a common phenotype of many complex diseases and has 1478 annotated genes in the HPO as of August 2020. We obtain 1896 genes related to ACSM, of which 1088 are true positive and 812 are false positive. The same case study method as “pneumonia” is used to get the case evidences as shown in Supplementary Table S2. Among the top 15 genes identified as false positives, we find supporting evidence for seven of them. “Angiogenic factor with G-patch and FHA domain1” (AGGF1) is a gene encoding a new angiogenic factor discovered by studying human congenital venous malformation limb hypertrophy syndrome. It is the only gene among the seven that is listed in HPO. The other six genes, although not present in HPO, have been proven to be closely related to ACSM. This also confirms the incomplete relationship between human genes and phenotypes in HPO.

## 4 Conclusion

In this article, we introduce a pre-training model SSLpheno, which is trained in a self-supervised manner on an attributed graph constructed from PPIs and gene ontology, and can predict disease phenotypes caused by genes lacking prior knowledge. We design a pretext task that selects positive and negative gene pairs based on cosine similarity, reconstruct their labels, and trains the optimal feature representation for downstream phenotype prediction. The experimental results demonstrate that our proposed model outperforms SOTA models in terms of performance. Further ablation studies suggest that each component in SSLpheno is necessary for our task. Multiple case studies from different perspectives also demonstrate the practicality of the SSLpheno model.

Our work shows the advantage of gene-enriched functional feature representation in phenotype prediction. However, during automatic encoding, the calculation of similarity between all pairs of nodes can be challenging for the computational performance of large networks. Overall, our proposed SSL method for phenotype prediction has the potential to meet the increasing demand for annotation data. In the future, we plan to explore methods that utilize existing annotation data to improve gene–phenotype prediction performance, providing new insights into the interpretation of genetic heterogeneity in diseases. Additionally, we aim to develop reasonable multi-source network feature fusion methods for gene–phenotype prediction as a future research direction.

## Supplementary Material

btad662_Supplementary_Data

## Data Availability

The code and datasets are available at https://github.com/bixuehua/SSLpheno.
