# Supplementary material for: SSLpheno: a self-supervised learning approach for gene–phenotype association prediction using protein–protein interactions and gene ontology data
Source: Bioinformatics. 2023 Nov 6;39(11):btad662. doi: 10.1093/bioinformatics/btad662 (PMC10666204; doi:10.1093/bioinformatics/btad662)
Supplement: btad662_Supplementary_Data [file btad662_supplementary_data.docx]

**SSLpheno: A Self-Supervised Learning Approach for Gene-Phenotype Association Prediction Using Protein-Protein Interactions and Gene Ontology Data**

**(Supplementary Data)**

Xuehua Bi^1,2^, Weiyang Liang^3^, Qichang Zhao^1^ and Jianxin Wang^1*^

^1^Hunan Provincial Key Lab on Bioinformatics, School of Computer Science and Engineering, Central South University, Changsha,410083, China. ^2^Medical Engineering and Technology College, Xinjiang Medical University, Urumqi, 830017, China. ^3^College of Information Science and Engineering, Xinjiang University, Urumqi, 830046, China.

* Contact: [jxwang@mail.csu.edu.cn](mailto:jxwang@mail.csu.edu.cn)

1 The distribution of datasets

We use two datasets HPO_2020 and DisGeNET. We divide the phenotype terms in datasets into four groups, ’11-30’, ’31-100’, ’101-300’, and ’>300’, according to the number of annotated genes. The annotations of each group in each dataset are calculated and the distribution is shown in Figure S1.

Figure S1: The proportion of phenotype annotation for each group in two datasets.

In the temporal validation, the training set consists of gene-phenotype associations released by the HPO database on November 15, 2019, while the test set consists of new associations added from November 15, 2019, to August 25, 2020. The distribution of average phenotype annotations per gene of the training set and test set in the temporal validation is shown in Figure S2.


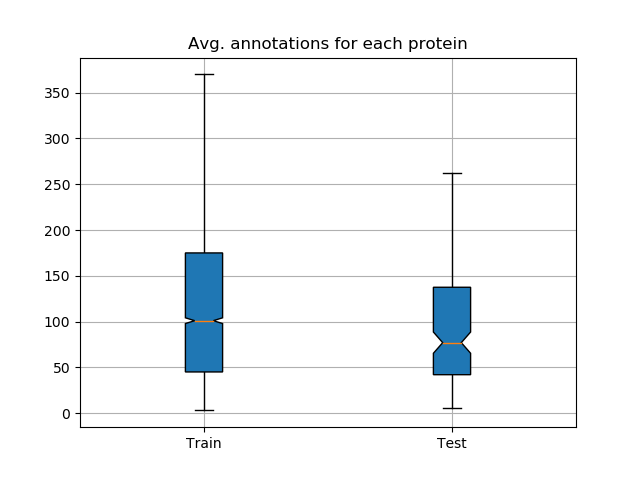
Figure S2: The distribution of average phenotype annotations per gene of training set and test.

2 The construction of GGAs

The performance of the model is affected by different GGAs. We use different PPI networks to build GGA. STRING, HumaNet, and GeneMANIA are three popular PPI networks. Here, we introduce the construction of GGAs by using the three PPIs. The statistical information and URL of GGAs are shown in Table S1.

(1) STRING

STRING is an integrated database that contains all known and predicted associations between proteins, including both physical interactions as well as functional associations [1]. This database collects evidence from databases of interaction experiments and annotated complexes/pathways, computational interaction precondition, systematic transfers of interaction across species, and text mining of scientific literature. In this work, we use STRING v11.0 (<https://string-db.org/>). We map the protein ID in STRING to the gene ID in Uniprot based on the mapping file in Uniprot.

(2) HumaNet

HumanNet is a human gene network that integrates inferred co-functional relations from diverse datasets, encompassing co-citation in PubMed articles, co-expression, protein-protein interaction, genetic interaction, protein domain co-occurrence, and genomic context similarity [2]. This network of human genes has been further expanded by including interaction between proteins evolutionarily conserved between humans and other organisms. In this work, we use the extended gene network by co-citation (named HumanNet-XC) to construct GGAs. We map the specific gene ID in GeneMANIA to the gene ID in Uniprot based on the mapping file in Uniprot.

(3) GeneMANIA

GeneMANIA is a website providing gene functional assays, which integrates data sets from publicly available co-expression, physical and genetic interaction, predicted protein interaction, pathway, and molecular interaction data [3]. The prediction server employs label propagation algorithms to find related genes and a linear regression-based algorithm to assign weights to networks. GeneMANIA provides gene-gene associations from the data either directly or using an in-house analysis pipeline to convert profiles.

Table S1. The detailed information of GGAs

| GGAs | Genes | Associations | URL |
| --- | --- | --- | --- |
| STRING v11.0 | 19080 | 11479004 | https://string-db.org/ |
| HumaNet-XC v3.0 | 18462 | 1125494 | https://staging2.inetbio.org/humannetv3/download.php |
| GeneMANIA 2018 | 19680 | 11749785 | https://genemania.org/ |

When we construct the GGAs, the edge between two genes is judged by a specific threshold. We set thresholds of 0.2, 0.3, and 0.4, respectively, to build the GGA and train SSLPheno based on STRING. The results are shown in Figure S3.

Figure S3: The model performances of STRING at different thresholds.


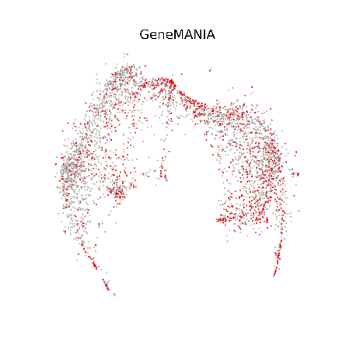
To explore the embeddings produced by SSLpheno with different GGAs, we embed these vector representations into a 2D space using t-SNE and visualize them in Figure S4. We highlight the associated genes with HP:0002206 with red dots. The features from STRING seem to be a little more concentrated than the features from HumanNet. The features are distributed entirely produced by GeneMANIA.


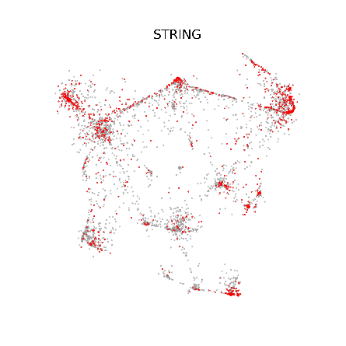

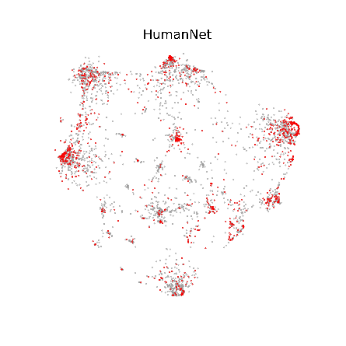
Figure S4: Visualization of learned embeddings on different GGAs, (A) STRING, (B) GeneMANIA, and (C) HumanNet. The embeddings are mapped to the 2D space using the t-SNE, where each point corresponds to a gene. The red dots highlight the genes associated with *Abnormality of the abdominal organs* (HP:0002012).

3 The search and optimization of the parameters

We perform a grid search for the hyperparameter tuning. Hyperparameter settings, search ranges, and optimization values are listed in Table S2.

Table S2: The hyper-parameters settings.

| Hyper-parameter | Search scopes | Optimized values |
| --- | --- | --- |
| Dimensions of PCA | [600,800,1000,1200,1400,1600] | 1000 |
| Number of filters | [2,3,4,5,6] | 4 |
| Hidden layers of the linear encoder | [1,2,3] | 1 |
| Units number in the hidden layer of the linear encoder | [512,1024,2048] | 2048 |
| The number of positive samples | [5000,10000,15000] | 10000 |
| Hidden layers of DNN | [2,3,4,5] | 3 |

We select six values, 600, 800, 1000, 1200, 1400, and 1600, of the PCA reduction dimension and conduct the experiment. The model performance results are shown in Figure S5.

Figure S5: The model performances in different PCA reduction dimensions.

We have conducted experiments with different α values and the results are shown in Figure S6.

Figure S6: The model performances under different *α* in the Laplacian smoothing filter.

We perform experiments with different gene representation dimensions, and when the value is set to 2048, the model prediction performance is optimal. The details are shown in Figure S7.

Figure S7: The model performances under different gene representation dimensions.

To further illustrate the helplessness of the ‘1-10’ group on model performance, we train our model using all the group data, and the results are shown in Figure S8.

Figure S8: The model performances on different groups.

We analyze the impact of ‘1-10’ group on model performance. The model is trained using the dataset with or without the ‘1-10’ group, respectively. The model performance on phenotype with more than 10 annotations is shown in Figure S9.

Figure S9: The model performances on ‘11-’ groups.

4 Case study on phenotypes

We conducted an analysis on *Abnormality of cardiovascular system morphology* (ACSM, HP0030680) and *Decreased activities of* *mitochondrial encoded respiratory chain complexes* (DAMRC, HP:0008972).

ACSM is a common phenotype of many complex diseases and has 1,478 annotated genes in the HPO as of August 2020. We obtained 1,896 genes related to ACSM, of which 1,088 are true positive and 812 are false positive. The results are shown in Table S3.

Table S3: The top 15 predicted false positive genes of *Abnormality of cardiovascular system morphology* with supporting literature.

| Rank | Gene name | Evidence | Rank | Gene name | Evidence |
| --- | --- | --- | --- | --- | --- |
| 1 | NSF | -- | 9 | SLC12A2 | -- |
| 2 | CASZ1 | PMID: 37182340 [4] | 10 | ALDH1A2 | PMID: 33565183 [9] |
| 3 | PRKCZ | PMID: 34151742 [5] | 11 | SLC2A1-AS1 | -- |
| 4 | ATP2B1 | PMID: 32155320 [6] | 12 | MS4A4E | -- |
| 5 | AGGF1 | PMID: 24893993 [7] | 13 | SLC2A1-AS1 | -- |
| 6 | MTX2 | -- | 14 | CDC5L | PMID: 32265825 [10] |
| 7 | GRM7 | -- | 15 | RPL17P35 | -- |
| 8 | WNT9B | PMID: 35697867 [8] |  |  |  |

Mitochondrial respiration is an ATP-generating process driven by a series of protein complexes that are located on the inner membrane of the mitochondria [11]. The damage caused by mitochondrial reactive oxygen species production is an essential molecular basis of various diseases including cancer. DAMRC is a phenotype discussed in GraphPheno, which is annotated with 36 genes in HPO 2020 and 50 genes in HPO 2023. We predict 178 genes related to DAMRC and the top 15 false positive genes are shown in Table S4.

Table S4: The top 15 predicted false positive genes of *Decreased activities of mitochondrial encoded respiratory chain complexes* with supporting literature.

| Rank | Gene name | Evidence | Rank | Gene name | Evidence |
| --- | --- | --- | --- | --- | --- |
| 1 | TUBA8 | -- | 9 | RARS1 | -- |
| 2 | AVP | -- | 10 | IL2RA | -- |
| 3 | AUTS2 | -- | 11 | RPL35 | -- |
| 4 | ND2 | -- | 12 | ATP2C1 | -- |
| 5 | TERF2IP | -- | 13 | AFG3L2 | PMID: [22252130](https://pubmed.ncbi.nlm.nih.gov/22252130) [12] |
| 6 | ANG | -- | 14 | KARS1 | -- |
| 7 | MLX | -- | 15 | RARS1 | -- |
| 8 | KIF1A | PMID: 30385166 [11] |  |  |  |

[1]Szklarczyk D, Kirsch R, Koutrouli M, et al. The STRING database in 2023: protein–protein association networks and functional enrichment analyses for any sequenced genome of interest[J]. Nucleic acids research, 2023, 51(D1): D638-D646.

[2] Kim C Y, Baek S, Cha J, et al. HumanNet v3: an improved database of human gene networks for disease research[J]. Nucleic acids research, 2022, 50(D1): D632-D639.

[3] Franz M, Rodriguez H, Lopes C, et al. GeneMANIA update 2018[J]. Nucleic acids research, 2018, 46(W1): W60-W64.

[4] Zhang F, Fu C, Deng Y, et al. Association of CASZ1 genetic variants with stroke risk in the Chinese population[J]. J Stroke Cerebrovasc Dis. 2023;32(8):107169.

[5] Infante T, Franzese M, Ruocco A, et al. ABCA1, TCF7, NFATC1, PRKCZ, and PDGFA DNA methylation as potential epigenetic-sensitive targets in acute coronary syndrome via network analysis[J]. Epigenetics. 2022;17(5):547-563.

[6] Song KY, Zhang XZ, Li F, Ji QR. Silencing of ATP2B1-AS1 contributes to protection against myocardial infarction in mouse via blocking NFKBIA-mediated NF-κB signalling pathway[J]. J Cell Mol Med. 2020;24(8):4466-4479.

[7] Liu Y, Yang H, Song L, et al. AGGF1 protects from myocardial ischemia/reperfusion injury by regulating myocardial apoptosis and angiogenesis[J]. Apoptosis, 2014, 19: 1254-1268.

[8] Pirruccello JP, Di Achille P, Nauffal V, et al. Genetic analysis of right heart structure and function in 40,000 people[J]. Nat Genet. 2022;54(6):792-803.

[9] Beecroft SJ, Ayala M, McGillivray G, et al. Biallelic hypomorphic variants in ALDH1A2 cause a novel lethal human multiple congenital anomaly syndrome encompassing diaphragmatic, pulmonary, and cardiovascular defects[J]. Hum Mutat. 2021;42(5):506-519.

[10] Zhang YF, Meng LB, Hao ML, Yang JF, Zou T. Identification of Co-expressed Genes Between Atrial Fibrillation and Stroke[J]. Front Neurol. 2020;11:184.

[11] Sreedhar A, Aguilera-Aguirre L, Singh KK. Mitochondria in skin health, aging, and disease. Cell Death Dis. 2020;11(6):444.

[12] Samanta D, Gokden M. PEHO syndrome: KIF1A mutation and decreased activity of mitochondrial respiratory chain complex[J]. Journal of Clinical Neuroscience, 2019, 61: 298-301.

[13] Hornig‐Do H T, Tatsuta T, Buckermann A, et al. Nonsense mutations in the COX1 subunit impair the stability of respiratory chain complexes rather than their assembly[J]. The EMBO journal, 2012, 31(5): 1293-1307.
